# Manganese-Implanted Titanium Modulates the Crosstalk between Bone Marrow Mesenchymal Stem Cells and Macrophages to Improve Osteogenesis

**DOI:** 10.3390/jfb14090456

**Published:** 2023-09-03

**Authors:** Kuicai Ye, Xianming Zhang, Li Shangguan, Xingdan Liu, Xiaoshuang Nie, Yuqin Qiao

**Affiliations:** 1 State Key Laboratory of High-Performance Ceramics and Superfine Microstructure, Shanghai Institute of Ceramics, Chinese Academy of Sciences, Shanghai 200050, China; kcyeh@mail.ustc.edu.cn (K.Y.);; 2 Center of Materials Science and Optoelectronics Engineering, University of Chinese Academy of Sciences, Beijing 100049, China; 3 School of Materials Science, Shanghai University, Shanghai 200444, China

**Keywords:** manganese, plasma immersion ion implantation and deposition, crosstalk, mBMSCs, macrophages

## Abstract

Manganese (Mn) is an essential micronutrient in various physiological processes, but its functions in bone metabolism remain undefined. This is partly due to the interplay between immune and bone cells because Mn plays a central role in the immune system. In this study, we utilized the plasma immersion ion implantation and deposition (PIII&D) technique to introduce Mn onto the titanium surface. The results demonstrated that Mn-implanted surfaces stimulated the shift of macrophages toward the M1 phenotype and had minimal effects on the osteogenic differentiation of mouse bone marrow mesenchymal stem cells (mBMSCs) under mono-culture conditions. However, they promoted the M2 polarization of macrophages and improved the osteogenic activities of mBMSCs under co-culture conditions, indicating the importance of the crosstalk between mBMSCs and macrophages mediated by Mn in osteogenic activities. This study provides a positive incentive for the application of Mn in the field of osteoimmunology.

## 1. Introduction

The process of osseointegration, in which chemical bonding between biomaterials and bone tissue occurs, is a highly complex and dynamic mechanism [1]. Inflammatory reactions play a crucial role in the recovery and reconstruction process of damaged bone tissue through a series of intricate cellular and molecular interactions [2,3]. Recently, modulating the immune responses has been recognized as a pivotal strategy to regulate or control the process of bone remodeling and offers a prospect and challenges for developments in bone biomedical devices [4].

Macrophages, one of the first cells to arrive at the site of injury, are present throughout the whole repair and regeneration process [5]. They can not only defend against pathogens but also, more importantly, secrete an array of cytokines and chemokines in response to certain stimuli and recruit other cells to promote inflammation [6]. Generally, macrophages are divided into at least two subgroups, the classically activated M1 phenotype and alternatively the M2 phenotype [7]. M1 macrophages, with a typical surface marker C-C chemokine receptor type 7 (CCR7, or CD197), usually feature a pro-inflammatory phenotype and tend to secrete pro-inflammatory cytokines including interleukin 6 (IL-6) and tumor necrosis factor-α (TNF-α), as well as chemokines such as C-C motif chemokine ligand 3 (CCL3), colony-stimulating factor 2 (CSF2), and C-C motif chemokine ligand 5 (CCL-5). And M2 macrophages, with the typical surface marker of mannose receptor 206 (CD206), are associated with anti-inflammatory responses that can accelerate the tissue healing process through anti-inflammatory cytokines such as interleukin 4 (IL-4), interleukin 10 (IL-10), and arginase 1 (Arg-1) [7,8]. This molecular mechanism suggests that biomaterials which can stimulate the macrophage polarization from M1 to M2 may enhance macrophage-mediated osteogenesis [9]. Thus, a lot of efforts have been spent on optimizing the physicochemical properties of biomaterials to induce M2 transition of macrophages in previous studies [10,11]. It is widely recognized that excessive inflammation can result in implant failure and complications such as peri-implantitis [12]. However, with the increasing understanding of macrophages, emerging evidence suggests that the osseointegration is intricately mediated by a complex interplay of various factors in the immune microenvironment, including not only M2 macrophages but also M1 macrophages [13,14,15]. And insufficient inflammation may also lead to slow osseointegration or poor bone-to-implant contact. Moreover, inconsistencies between in vitro and in vivo studies are not uncommon, indicating that the mechanisms which modulate the materials’ capacity to regulate osteogenic activities in the immunological microenvironment are not well understood [16]. Therefore, it is necessary to investigate the role of the interactions between immune- and bone-related cells mediated by biomaterials in osteogenesis.

Some nutrient elements have been found to play key roles in bone regeneration and remodeling and also elicit significant immune responses [17,18,19,20]. For example, copper-incorporated biomaterials (bioactive glass, ceramics, and titanium alloys) have been found to improve osteogenic activities and angiogenic properties [21,22]. In a recent study, Huang et al. showed that a Cu-containing micro/nano-topographical bio-ceramic surface promoted macrophages to the M1 pro-inflammatory phenotype by activating Cu-transport signaling, which can enhance the phagocytic ability on bacteria [23]. Among these elements, Mn also serves as a cofactor in various enzymatic reactions and is crucial for the proper functioning of many enzymes, such as manganese superoxide dismutase (Mn-SOD) [24]. And comparatively, Mn plays a more significant role in the modulation of immune responses rather than in bone metabolism [18,25]. Increasing evidence has mounted to suggest that Mn is an immunostimulatory agent which augments the inflammatory response through the cGAS-STING signaling pathway [26]. Although some Mn-incorporated biomaterials have been reported to stimulate the osteogenic differentiation of pre-osteoblasts or mesenchymal stem cells, there is still little evidence on how Mn-incorporated biomaterials affect osteogenic activities in the immune microenvironment [27,28,29]. Therefore, further investigations are focused on the manganese-mediated regulation of the immune system in the context of osteogenic effects.

In this study, we employed the plasma immersion ion implantation and deposition (PIII&D) technique to introduce Mn onto the surface of the titanium. This method is superior because it can incorporate elements into various medical devices with irregular shapes and minimize the effects of other confounding factors on the biological properties [30]. The Raw264.7 macrophage cell line was utilized to investigate the impact of materials on cellular immune responses in our study due to its ease of culture and strong phenotypic stability [31]. Raw264.7 cells can elicit a robust and well-known inflammatory response upon challenges with increasing dosages of stimulants, such as lipopolysaccharide (LPS), which is often employed to screen biomaterials and to predict their potential in regulating immune responses [31]. This may ultimately activate a series of events, including the production of inflammatory and anti-inflammatory cytokines, which can be used as biomarkers to screen for possible anti-inflammatory and immunomodulatory compounds or biomaterials [32]. Both mono- and co-culture experiments of Raw264.7 cells and mBMSCs were conducted to deepen the understanding of the crosstalk between macrophages and mBMSCs mediated by Mn-implanted surfaces.

## 2. Materials and Methods

### 2.1. Preparation of Mn-Implanted Titanium

In this study, two distinct sizes of titanium plates (Cp Ti, TA1, purity > 99.85%) were employed: 20 mm × 10 mm × 1 mm plates were utilized for zeta potential measurement, and 10 mm × 10 mm × 1 mm plates were used in the remaining experiments. The titanium plates were initially polished with silicon carbide abrasive papers and then ultrasonically washed with acetone and alcohol. Prior to plasma immersion ion implantation and deposition (PIII&D), titanium plates were cleaned using radio frequency argon ions for 10 min at a bias of 800 V. The Mn cathode (purity > 99.9%) was obtained from Qi Jin New Material Co., Ltd. (Quanzhou, China). Then, Mn ions were implanted onto the pretreated titanium surface at 15 kV for 60 min (denoted as Mn60) and 120 min (denoted as Mn120). During PIII&D, the sample stage was cooled by circulating water to keep the sample temperature at 25 °C. The specific parameters are outlined in Table 1.

### 2.2. Characterization

#### 2.2.1. Surface Structure and Physicochemical Characterization

Field emission scanning electron microscopy (FE-SEM; Magellan 400, Hillsboro, OR, USA) was used to analyze the surface morphologies of materials at a 5 kV acceleration voltage. The elemental valences, contents, and distribution of Mn-implanted surfaces were analyzed using X-ray photoelectron spectroscopy equipped with a monochromatic Al Kα source (XPS; K-Alpha, Thermo Scientific, Waltham, MA, USA). The nano-hardness and elastic modulus of Mn-implanted surfaces were determined using a nanoindenter (G200, Agilent, Santa Clara, CA, USA), and the average value of each sample was obtained from five measurements. Each experiment was independently performed twice.

#### 2.2.2. Surface Wettability

The surface wettability was measured with a surface contact angle meter (SL200B, Solon, Shanghai, China). The water droplets were captured and analyzed after 2 μL of ultrapure water was vertically dropped onto surfaces. The experiment was performed twice independently with three replicates.

#### 2.2.3. Dynamic Potential Polarization Test

An electrochemical workstation (CHI760, Shanghai Chenhua, Shanghai, China) was used for electrochemical analysis, and the electrolyte was a 0.9% NaCl solution. The measurement was performed by utilizing a conventional and electrochemical cell with three-electrodes. The reference, counter, and working electrodes were a saturated calomel electrode, graphite, and a test sample, respectively. The scanning rate was set as 0.01 V/s. The experiment was performed twice independently.

#### 2.2.4. Surface Zeta Potential

The surface zeta potential of the plates was measured using a SurPASS 3 electric analyzer (AntonPaar, Graz, Austria). Two 20 mm × 10 mm × 1 mm samples were put on the test cell. The space between two parallel samples was set to 100 ± 5 μm to guarantee that the electrolyte (0.001 mol/L KCl solution) went through the gap. The pH of the measurement was set from 10.0 to 5.0 and adjusted by automatic machine titration of 0.05 M HCl. The final value was taken as the average of four measurements for each measurement point. The calculation formula was determined according to Helmholtz–Smoluchowski equation [33]:

$$\zeta =\frac{dU}{dP}\frac{\eta }{\varepsilon \varepsilon_{0}}K$$

in which ζ is the zeta potential, *dU/dP* stands for the slope of the streaming potential versus differential pressure, and η, ε, $\varepsilon_{0},$ and K denote the electrolyte viscosity, vacuum permittivity, dielectric constant of the electrolyte, and electrical conductivity, respectively.

#### 2.2.5. Concentration of Released Mn ions

Mn-implanted samples were immersed in 5 mL PBS (phosphate-buffered saline, pH = 7.4) and kept at 37 °C. At the end of 1, 4, 7, and 14 days, PBS solution was collected and replaced with fresh 5 mL PBS. The Mn ion concentration was determined using inductively coupled plasma mass spectrometry (ICP-MS, X Series 2, Thermo Scientific, Waltham, MA, USA). Each group of samples was tested in duplicate.

### 2.3. Biological Evaluations

#### 2.3.1. Cell Culture

The effects of different surfaces on the behavior of macrophages were evaluated using mouse mononuclear macrophage leukemia cells (Raw264.7, obtained from Cell Bank of the Chinese Academy of Sciences, Shanghai, China), which were cultured in DMEM medium (high glucose, Gibco, Waltham, MA, USA) supplemented with 15% fetal bovine serum (FBS, Gibco, Waltham, MA, USA) and 1% penicillin/streptomycin (100 U/mL, Gibco, Waltham, MA, USA) at 37 °C in a humidified environment of 5% CO_2_. Cells were passaged at a 1:3 ratio every three days and maintained up to 10 passages. 

The osteogenic activity of various samples was evaluated using mouse bone marrow mesenchymal stem cells (mBMSCs, obtained from Shanghai Zhong Qiao Xin Zhou Biotechnology Co., Ltd., Shanghai, China). The mBMSCs were maintained in a basal medium composed of Mesenchymal Stem Cell Medium (MSCM, Sciencell, Carlsbad, CA, USA), 25 mL of FBS (Sciencell, Carlsbad, CA, USA), 5 mL of mesenchymal stem cell growth supplement (Sciencell, Carlsbad, CA, USA), and 5 mL of penicillin/streptomycin solution (Sciencell, Carlsbad, CA, USA). For osteogenic differentiation of mBMSCs, the osteogenic differentiation media were composed of DMEM medium (high glucose, Gibco, Waltham, MA, USA), 15% FBS (Gibco, Waltham, MA, USA), 1% penicillin/streptomycin serum (100 U/mL, Gibco, Waltham, MA, USA), 100 nM dexamethasone (Sigma-Aldrich, Burlington, MA, USA), 50 μg/mL L-ascorbic acid (Sigma-Aldrich, Burlington, MA, USA), and 10 mM β-glycerophosphate (Sigma-Aldrich, Burlington, MA, USA). The cells were passaged at a ratio of 1:3 every three days in a humidified atmosphere of 5% CO_2_ at 37 °C and were used in this study within 5 passages. 

In this work, co-culture experiments were performed to investigate the interaction effects between Raw264.7 cells and mBMSCs. The experimental methods were in reference to the literature report [34]. The experimental schematic is shown in Figure S1. Firstly, Raw264.7 cells and mBMSCs were incubated on different samples (four plates per group) for 1 day and 7 days, respectively. Then, 2 samples from each group, which had been seeded with Raw264.7 cells and mBMSCs, were put in the same well of a 6-well plate to co-culture for an additional 3 days. An osteogenic differentiation medium was used in the co-culture condition. At the end of the incubation time, samples seeded with Raw264.7 cells and mBMSCs were separately transferred to different new plates for further biological evaluations.

#### 2.3.2. Cell Proliferation and Viability

AlamarBlue^TM^ (Thermo Fisher Scientific, Waltham, MA, USA) was used to detect cell viability. At the end of different incubation time points, the medium was replaced by 500 μL of fresh culture medium containing 10% AlamarBlue^TM^ and incubated at 37 °C for 2 h. Then, medium aliquots (100 μL per well) were transferred to a black 96-well plate to measure the fluorescence intensity (E_x_ = 560 nm, E_m_ = 590 nm) using a multifunctional microplate reader (Cytation 5, BioTek, Winooski, VT, USA). 

Specifically, Raw264.7 cells were inoculated into a 24-well plate at a density of 1 × 10^5^ cells per well, and cell viability was measured at 4 h and 1 and 4 days. The mBMSCs were inoculated into a 24-well plate at a density of 2 × 10^4^ cells per well, and the measured time points were at 1, 4, and 7 days. Four parallel samples were set in each group. The experiment was performed twice independently for each experiment.

#### 2.3.3. Cell Morphology 

Raw264.7 cells were seeded at a density of 1 × 10^5^ cells per well on samples (four replicates) in a 24-well plate. The cells were cultured for 4 days. Then macrophages were washed with PBS and fixed with 2.5% glutaraldehyde at 4 °C overnight. Following this, the cells were dehydrated in a series of ethanol solutions (30, 50, 75, 90, 95%, and anhydrous ethanol, *v*/*v*) for 10 min each and dried in the hexamethyl disilylamine/ethanol solution series (1: 2, 1: 1, 2: 1 and 3:0, *v*/*v*). All samples were treated with gold spraying (120 s). Then, the cell morphologies were observed using SEM (accelerating voltage: 5 kV, S-3400 N Type I, Hitachi, Tokyo, Japan).

mBMSCs were seeded at a density of 2 × 10^4^ per well in 24-well plates and incubated for 4, 12, and 24 h. After being fixed with 4% paraformaldehyde overnight at 4 °C in the dark, the cells were permeabilized with 0.1% Triton X-100 (Amresco, Solon, OH, USA) for 2 min, blocked with 1% Bovine Serum Albumin (BSA, Sigma, Saint-Louis, MO, USA) for 30 min, and subsequently stained with rhodamine phalloidin (Sigma, Saint-Louis, MO, USA) at room temperature for 1 h and 4′,6-diamidino-2-phenylindole (DAPI, Sigma-Aldrich, Burlington, MA, USA) for an additional 3 min. Finally, the F-actin of mBMSCs was observed using fluorescence microscopy (IX71, Olympus, Tokyo, Japan).

#### 2.3.4. Immunofluorescence Staining of Macrophages

Mannose receptor 206 (CD206, M2 marker, in red) and inducible nitric oxide synthase (iNOS, M1 marker, in green) were selected to evaluate the polarization state of macrophages. Firstly, macrophages were cultured on different sample surfaces at a density of 1 × 10^5^ per well for 4 h and 1 and 4 days. At the end of incubation, cells were washed with PBS three times, fixed with 4% paraformaldehyde (PFA, Sigma, Saint-Louis, MO, USA) at 4 °C for 10 min, permeabilized by 0.1% Triton X-100 (Amresco, Solon, OH, USA) for 2 min, and blocked with 3% BSA (Sigma, Saint-Louis, MO, USA) in PBS for 1 h. Then, the cells were incubated with rabbit anti-mouse iNOS (1: 50, Cell Signaling Technology, Beverly, MA, USA) and goat anti-mouse CD206 (1:40, R&D Systems, Minneapolis, MN, USA) at 4 °C overnight. Subsequently, the cells were incubated with secondary antibodies donkey anti-rabbit IgG H&L Alexa Fluor 488 (1:200, Invitrogen, Thermo Fisher Scientific, Waltham, MA, USA) and donkey anti-goat IgG H&L Alexa Fluor 594 (1: 200, Abcam, Cambridge, UK) for 2 h at room temperature. The nuclei of the cells were stained with DAPI for 3 min in the dark. The immunofluorescence staining was visualized using a fluorescence microscope (IX71, Olympus, Tokyo, Japan). The experiment was performed twice independently.

#### 2.3.5. Flow Cytometry Analysis of Macrophages

C-C chemokine receptor type 7 (CCR7) and CD206 were used to characterize M1 and M2 phenotypes of macrophages, their expression levels were detected using flow cytometry, and F4/80 was selected as the marker of Raw264.7 cells. Macrophages were seeded at a density of 1 × 10^5^ per well on samples (four plates per replicate and three replicates per group) in a 24-well plate for 4 days. Then, the cells were detached by trypsin-EDTA (0.05%), washed with PBS, and resuspended in PBS. Single-stained cells served as compensation controls, while unstained cells were used to set the negative gate. The resuspended cells were blocked with mouse Fc block (BD Pharmingen, San Diego, CA, USA) for 10 min at room temperature. Then, the blocked cells were sequentially stained with PE/Cy7-labeled anti-mouse F4/80 antibody (BD Pharmingen, San Diego, CA, USA), APC-labeled anti-mouse CD206 antibody (Biolegend, San Diego, CA, USA), and PE-labeled anti-mouse CD197 (CCR7) antibody (Biolegend, San Diego, CA, USA) for 30 min at 4 °C in the dark. Then, they were washed and resuspended with PBS and detected on the flow cytometer (Celesta, BD Biosciences, San Jose, CA, USA). 

#### 2.3.6. Real-Time Quantitative Polymerase Chain Reaction (RT-qPCR) Analysis

RT-qPCR was utilized to measure the immune- and osteogenic-related gene expression. Four samples per group were placed in a 24-well plate and then seeded with Raw264.7 cells at a density of 1 × 10^5^ cells per well and mBMSCs at a density of 2 × 10^4^ cells per well. Raw264.7 cells were cultured for 4 days and mBMSCs were cultured for 10 days. At the end of incubation, samples were moved to a fresh six-well plate, washed with PBS twice, and adhered cells were blown off with 1 mL Trizol^TM^ (Invitrogen, Thermo Fisher Scientific, Waltham, MA, USA). Complementary DNA (cDNA) was synthesized from total RNA using a transcriptor first-strand cDNA synthesis kit (Roche, Basel, Switzerland) according to the manufacturer’s protocol. RT-qPCR was performed on the LightCycler^®^ 480 system II (Roche, Basel, Switzerland) utilizing SYBR Green I Master (Roche, Basel, Switzerland). The level of each target gene was calculated using the 2^−ΔΔCt^ method, and GAPDH was used as a reference for normalization. The primers used in RT-qPCR are given in Table S1 and were obtained from BioTNT (Shanghai, China). Each group of samples was analyzed in triplicate. 

#### 2.3.7. Alkaline Phosphatase (ALP) Activity of mBMSCs

mBMSCs were seeded on different samples (four replicates per group) at a density of 2 × 10^4^ cells per well in 24-well plates. The cells were cultured in osteogenic differentiation medium for 10 days. Cells were lysed for 40 min on ice using a lysis buffer containing 1% protease inhibitor (Sigma-Aldrich, Burlington, USA), 1% IGEPAL CA-630 (Beyotime-Biotech Co., Shanghai, China), 10 mM Tris-HCl (Sinopharm, Shanghai, China, pH = 7.5), and 1 mM MgCl_2_ (Sinopharm, Shanghai, China) [35]. Then, cell lysates were centrifuged at 8000 rpm for 10 min at 4 °C, and the supernatant was collected. After that, p-nitrophenyl phosphate (*p*NPP) (Sigma-Aldrich, Burlington, MA, USA) was added and incubated for 30 min at 37 °C. Then, 1 M NaOH (Sinopharm, Shanghai, China) solution was added to terminate the reaction. The ALP activity was measured using a multifunctional microplate reader (Cytation 5, BioTek, Winooski, VT, USA) at an absorbance of 405 nm for detecting the production of *p*-nitrophenol [36]. Total intracellular protein concentration was quantified using a bicinchoninic acid (BCA) kit (Thermo Fisher Scientific, Waltham, MA, USA) and used to normalize the ALP activity. 

For ALP staining, a 5-bromo-4-chloro-3-indolylphosphate/nitrobluetetra-zoliumchloride (BCIP/NBT) alkaline phosphatase color development kit (Beyotime-Biotech Co., Shanghai, China) was used. After incubation for 10 days, cells were treated with BCIP/NBT working solutions for 2 h in the dark and washed twice with ultrapure water. A fluorescent microscope (IX71, Olympus, Tokyo, Japan) was used to observe the stained cells. 

#### 2.3.8. Collagen Secretion of mBMSCs

mBMSCs were seeded on different samples (four replicates per group) at a density of 2 × 10^4^ cells per well in a 24-well plate and were cultured in osteogenic differentiation medium for 10 days. After finishing the culture, cells were fixed with 4% PFA (Sigma, Saint-Louis, MO, USA) for 10 min and stained with 0.1% Sirius Red solution (dissolved in saturated picric acid) for 18 h [34]. Afterward, the cells were washed with 0.1 M acetic acid to remove the excess dye. Then, the images were captured using a fluorescent microscope (IX71, Olympus, Tokyo, Japan) under a brightness field. The quantitative results were obtained by dissolving the stain in a mixed solution (0.5 mL per well, 0.2 M NaOH: methanol = 1:1, *v*/*v*), and the absorbance of the stain was measured at 540 nm using a multifunctional microplate reader (Cytation 5, BioTek, Winooski, VT, USA). The experiment was performed twice independently.

#### 2.3.9. Extracellular Matrix Mineralization (ECM) of mBMSCs

mBMSCs were seeded at a density of 2 × 10^4^ cells per well on samples (four replicates) in 24-well plates and cultured in an osteogenic differentiation medium for 10 days. The culture medium was changed every 2 days. After that, the cells were fixed with 75% ethanol solution for 1 h and subsequently stained with 40 mM Alizarin Red (pH = 4.2) for 10 min. The excess dye was removed with distilled water before taking images with a fluorescent microscope (IX71, Olympus, Tokyo, Japan) under a brightness field. An eluent (0.5 mL per well, 10% cetylpyridinium chloride (CPC) diluted in 10 mM sodium phosphate, pH = 7) was applied to dissolve the stain, and the absorbance of the stain at 620 nm was evaluated to quantify the ECM mineralization using a multifunctional microplate reader (Cytation 5, BioTek, Winooski, VT, USA). The experiment was performed twice independently.

### 2.4. Statistical Analysis

Statistical analysis was conducted utilizing GraphPad Prism 6.0 (GraphPad Software, La Jolla, CA, USA). Quantitative data were presented as the mean ± standard deviation (SD). The significance of differences (*p*-value) was analyzed through the use of one-way analysis of variance (ANOVA), two-way ANOVA, and Tukey’s multiple comparison tests. A *p*-value less than 0.05 was considered statistically significant and denoted by the symbol “*”, a *p*-value less than 0.01 was denoted by “**”, and a *p*-value less than 0.001 was denoted by “***”. The "ns" means not statistically significant.

## 3. Results

### 3.1. Surface Characteristics

**Figure 1:**
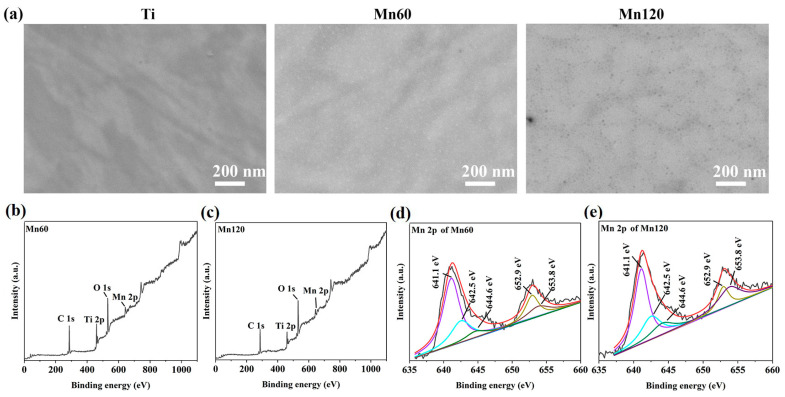
(**a**) SEM images of the surface morphologies of various samples; (**b**) XPS full spectrum of Mn60; (**c**) XPS full spectrum of Mn120; (**d**) XPS high-resolution spectrum of Mn 2p obtained from Mn60; (**e**) XPS high-resolution spectrum of Mn 2p obtained from Mn120.

The SEM images of Mn-implanted surfaces are shown in Figure 1. After the polishing process, the Ti surface was structurally flat. No obvious fluctuation in topography was observed. After Mn implantation, there were no visible structural changes on the surfaces of Mn60 and Mn120. 

XPS was conducted to determine the elemental composition of the sample surfaces. The relative amounts of Mn were 3.20% and 4.09% for Mn60 and Mn120, respectively (Table 2). Due to the interference of adsorbed C on the surfaces, the amount of implanted Mn in the two samples cannot be compared directly according to their relative elemental percentages. The Mn 2p peaks could be found from Mn-implanted surfaces (Figure 1b,c). As shown in the Mn 2p high-resolution spectra (Figure 1d,e), the peaks at 642.5 eV and 653.8 eV correspond to the 2p_3/2_ and 2p_1/2_ peaks of MnO_2_, respectively [37], the peaks at 641.1 eV and 652.9 eV correspond to the 2p_3/2_ and 2p_1/2_ peaks of MnO_x_/Mn, respectively, and the peak at 644.6 eV is considered to be the broad satellite peak of MnO_x_ [38,39]. The results demonstrated that Mn was present on the surface in the form of manganese oxides.

**Figure 2:**
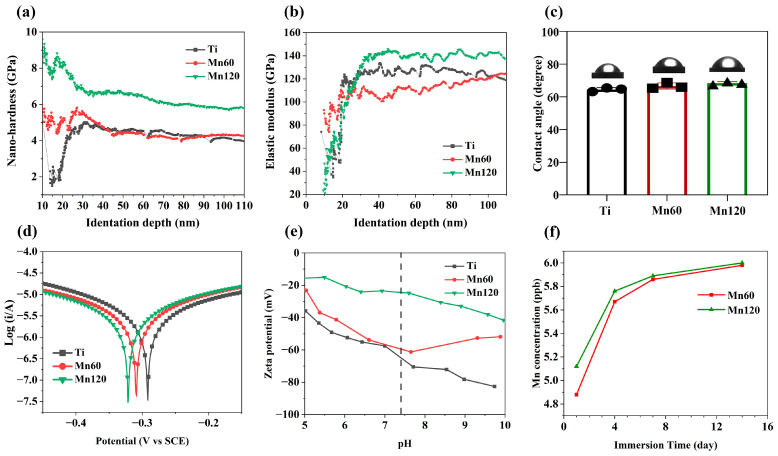
Physicochemical characterization of Mn-implanted surfaces: (**a**) nano-hardness curves of various samples; (**b**) elastic modulus curves of various samples; (**c**) water contact angles measured in various samples; (**d**) polarization curves of various samples; (**e**) surface zeta potential of various samples; (**f**) the concentrations of Mn ions in PBS solution.

When performing PIII&D, Mn implantation and deposition took place simultaneously on the surfaces of Mn60 and Mn120 by modulating the pulse width of the cathode to be bigger than that of the target (Table 1), which may affect the mechanical properties of titanium. In this study, changes in nano-hardness and elastic modulus within the 110 nm region were measured (Figure 2a,b). As shown in Figure 2a, compared to pure titanium, the surface nano-hardness of the Mn120 exhibited an obvious increase compared to titanium within the 110 nm region, while the nano-hardness of Mn60 only improved within the range of 15 nm to 30 nm. Figure 2b illustrates the variation in the specific elastic modulus on the modified surfaces. Different from the tendency of Mn implantation on nano-hardness, the surface elastic modulus of Mn60 and Mn120 exhibited a slight variation (Figure 2b). 

The relative surface wettability of the coatings was determined according to the water contact angle measurement. As shown in Figure 2c, Mn implantation did not alter the contact angles of Mn60 and Mn120 compared to Ti. Figure 2d and Table 3 exhibit Tafel curves and relevant data for different samples. The corrosion potentials of the Mn60 and Mn120 displayed a slight negative shift (0.017 eV for Mn60 and 0.035 eV for Mn120).

The role of surface charge in regulating cell responses has been extensively reported. As shown in Figure 2e, the zeta potentials of Mn-implanted surfaces presented a decreasing trend in a pH range from 5.0 to 7.4. At a pH of 7.4, Mn60 (−57.9 mV) and Mn120 (−24.3 mV) presented a more positive value than Ti (−61.2 mV). Previous studies have reported that a high concentration of manganese ions was detrimental to cells and can also influence the behavior of immune cells. The accumulative Mn ion release is shown in Figure 2f. During a 14-day period, the accumulative profiles of Mn60 and Mn120 showed similar Mn ion release characteristics in PBS, which include a burst release followed by a low-level continuous release. Although Mn120 displayed a higher release amount, both Mn-implanted surfaces released Mn ions at ppb level (0–10 ppb). This release profile can reflect that the amount of Mn implanted in Mn120 was higher than that implanted in Mn60.

### 3.2. Effects of Mn Implantation on Cellular Immune Response

**Figure 3:**
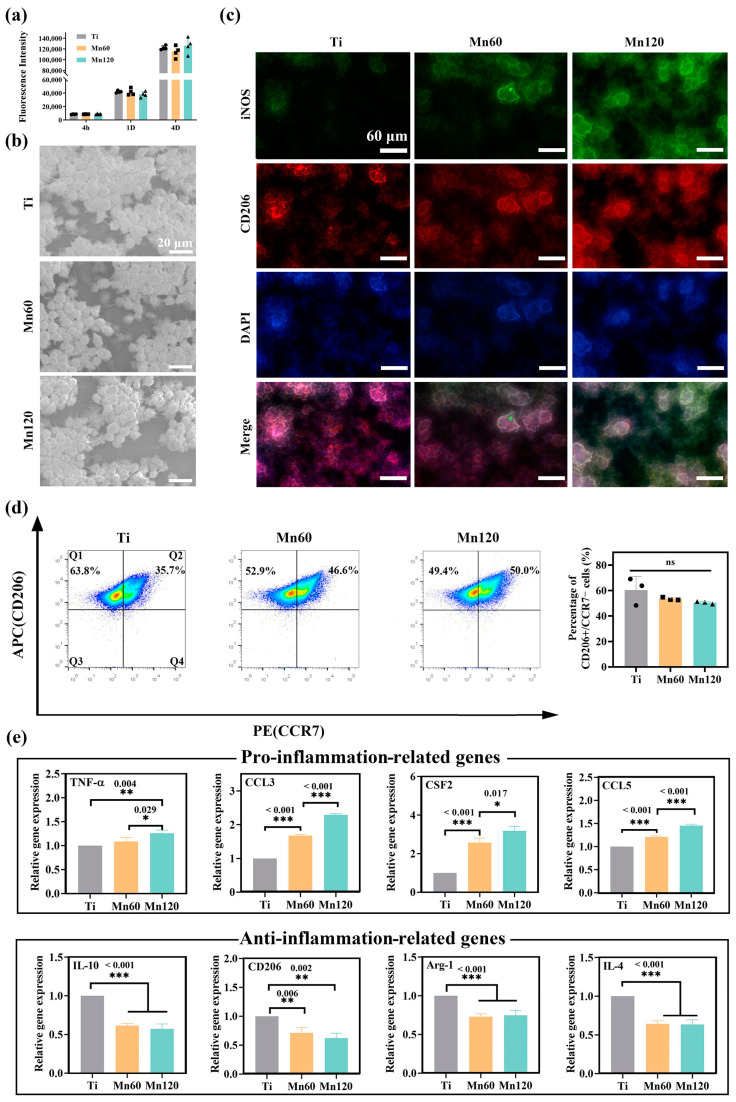
Mono-cultured Raw264.7 cells on various samples after incubation for 4 days: (**a**) cell viability; (**b**) SEM morphology; (**c**) immunofluorescent staining images; (**d**) flow cytometry analyses; (**e**) relative mRNA expression levels of the related genes.

The effects of Mn-implanted surfaces on macrophage polarization and plasticity were investigated using Raw264.7 cells. The cell viability results showed that Mn implantation did not exert any negative effects on cell proliferation (Figure 3a). When cultured for 4 days, round macrophages with long filopodia grew in grape-like clusters on all surfaces (Figure 3b), suggesting that Mn implantation had no obvious effects on the adhesion and spreading of macrophages. 

The polarization state and activation process of macrophages were evaluated at both gene and protein level using immunofluorescent staining, flow cytometry, and RT-PCR methods. As shown in Figure 3c, macrophages cultured on the Mn120 sample for 4 days expressed higher levels of iNOS (green) and similar levels of CD206 (red) compared to those on Ti and Mn60. The proportion of the M1 and M2 phenotypes of macrophages was quantitatively measured using flow cytometry (Figure 3c). The results demonstrated that the average proportion of CD206^+^ / CCR7^−^ (M2) macrophages of Ti was 60.4 ± 10.8%. The proportion of CD206^+^ / CCR7^−^ (M2) macrophages of Mn60 and Mn120 was 53.4 ± 1.2% and 50.5 ± 1.0%, respectively. Although there was no significant difference among the three groups, there was still a decreasing trend of M2 macrophages on Mn-implanted surfaces compared to those on Ti. To further investigate the influence of Mn implantation on macrophages, the expressions of macrophage marker genes were quantified using RT-PCR after being cultured for 4 days (Figure 3d). Furthermore, growth factors, cytokines, and chemokines released in the microenvironment may force macrophages to undergo substantial phenotypic and functional changes. Cytokines such as C-C motif chemokine ligand 3 (CCL3), colony-stimulating factor 2 (CSF2), and C-C motif chemokine ligand 5 (CCL5) have been reported to induce a transition in the phenotype of macrophages into a pro-inflammatory type [40,41,42,43]. The RT-qPCR results showed that the mRNA expressions of pro-inflammatory-related gene tumor necrosis factor-α (TNF-α) and cytokines including CCL3, CSF2, and CCL5 were significantly upregulated (Figure 3e) on different surfaces with the trend Mn120 > Mn60 > Ti, while those of anti-inflammatory-related genes, including interleukin 10 (IL-10), CD206, arginase 1 (Arg-1), and interleukin 4 (IL-4), were downregulated. 

### 3.3. Little Effects of Mn Implantation on Osteogenic Differentiation

**Figure 4:**
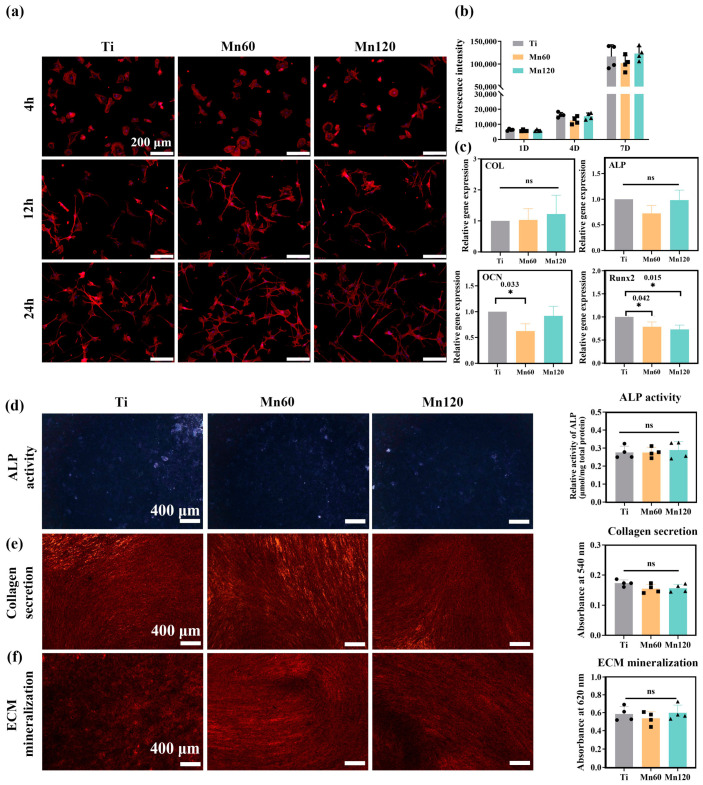
Osteogenic activity of mono-cultured mBMSCs on various samples after incubation for 10 days: (**a**) images of F-actin filaments; (**b**) cell viability assay; (**c**) relative mRNA expression levels of the related genes; (**d**) ALP staining images and quantitative results; (**e**) collagen secretion and the quantitative results; (**f**) extracellular matrix mineralization and the quantitative results.

It is well known that cell adhesion provides cells with the ability to sense the mechanical properties of the substratum, especially surface elastic modulus [44]. Figure 4a shows the F-actin filaments of mBMSCs on different surfaces. After seeding for 4 h, F-actin spread and most of the cells had a round shape, while a small portion of the cells exhibited star or spindle shapes. After seeding for 12 h and 24 h, a majority of cells were stretched out lengthwise, and linear F-actin filaments with a ruffle formation were observed on both Ti and Mn-implanted surfaces. There was no obvious difference in F-actin and the shape of mBMSCs among surfaces, indicating that the variation in elastic modulus between Ti and Mn120 did not exhibit an obvious impact on the process of cell attachment. When cultured on these surfaces for 4 or 7 days, there was no obvious change in the proliferation of mBMSCs, and the difference was not statistically significant (*p* > 0.05) (Figure 4b). 

To investigate the influence of Mn implantation on osteogenic differentiation, RT-qPCR was used to quantify the mRNA expressions of osteogenic-related genes, including early markers, such as alkaline phosphatase (ALP), type I collagen (COL) and runt-related transcription factor 2 (Runx2), as well as later marker osteocalcin (OCN) (Figure 4c). Runx2 is a key bone-specific transcription factor which plays an essential role in the commitment of pluripotent mesenchymal cells to the osteoblastic lineage [45,46]. Comparatively, the mRNA expressions of OCN and Runx2 were obviously downregulated on Mn60 compared to on Ti (*p* < 0.05), and Mn120 with a higher amount of Mn did not further enhance the decreasing trend. 

The osteogenic differentiation was further examined at the protein level. As shown in Figure 4d–f, the qualitative and quantitative results demonstrated that Mn implantation exhibited neglected effects on ALP activity, collagen secretion, and ECM mineralization (*p* > 0.05). These observations demonstrated that the osteogenic activities of mBMSCs were barely unaffected by Mn ions at lower concentrations (0–10 ppb).

### 3.4. Positive Immunomodulatory Effects of Mn Implantation on Osteogenic Differentiation

**Figure 5:**
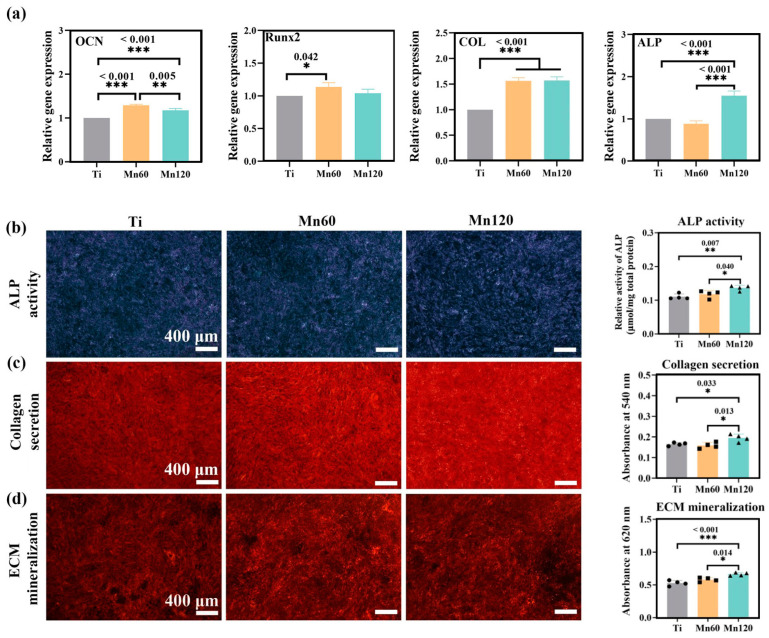
Osteogenic activities of mBMSCs on various samples after co-culturing for 10 days: (**a**) relative mRNA expression levels of osteogenic-related genes; (**b**) ALP staining and quantitative results; (**c**) collagen secretion staining and quantitative results; (**d**) extracellular matrix mineralization staining and the quantitative results.

As Mn plays an important role in immunological functions, and the immune response has a far-reaching impact on osteogenic activities, it is possible that Mn can exert an effect on osteogenic activities through its modulation of macrophages. After mBMSCs were co-cultured with Raw264.7 cells for 4 days, the RT-qPCR data revealed that Mn ions upregulated the mRNA expressions of osteogenic-related genes, such as OCN, Runx2, ALP, and COL (Figure 5a). Furthermore, ALP activities, collagen secretion, and ECM mineralization were also significantly promoted by Mn120 under co-culture conditions (Figure 5b–d). 

**Figure 6:**
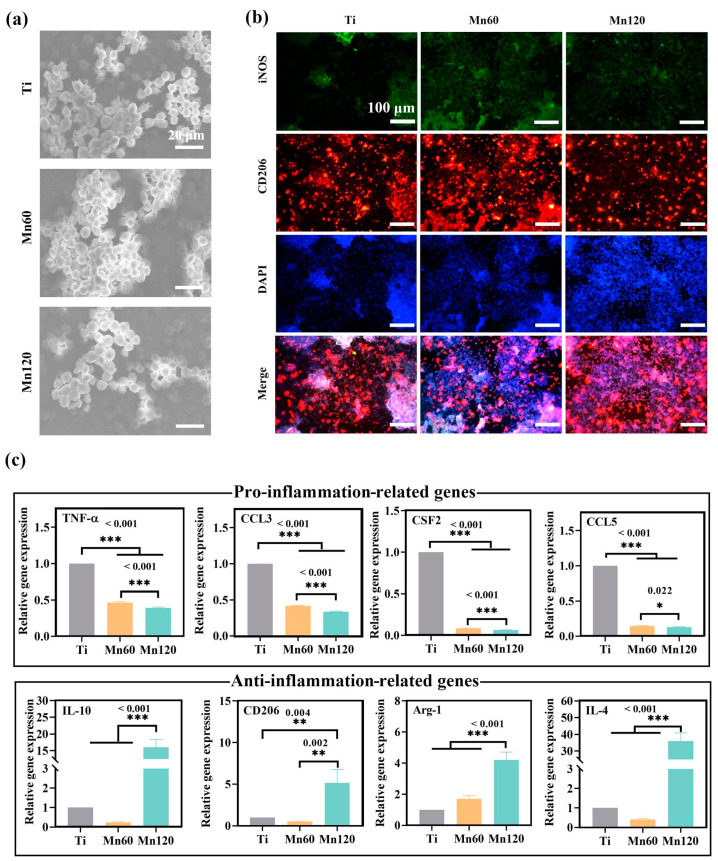
Raw264.7 cells on various samples under co-culture condition for 4 days: (**a**) SEM morphology; (**b**) immunofluorescent staining images; (**c**) relative mRNA expression levels of the related genes.

The effects of Mn-induced osteogenic activities on macrophages were also investigated under co-culture conditions for 4 days. As shown in Figure 6a, Raw264.7 cells had a round morphology on both Ti and Mn-implanted surfaces, which were similar to the morphology of macrophages under mono-culture conditions. The phenotype of Raw264.7 cells was identified using immunofluorescent staining and RT-qPCR experiments (Figure 6b,c). Immunofluorescent staining results showed that the macrophages on all surfaces expressed similar intensities of M1 marker iNOS (green) and M2 marker CD206 (red). The mRNA expressions of inflammatory markers and cytokines were evaluated using RT-qPCR. As shown in Figure 6c, the Mn-implanted surface Mn120 led to a remarkably increased mRNA expression of anti-inflammatory markers, including interleukin 10 (IL-10), CD206, arginase 1 (Arg-1), and interleukin 4 (IL-4). At the same time, both Mn60 and Mn120 significantly downregulated the expressions of TNF-α, CCL3, CSF2, and CCL5.

## 4. Discussion

The modulatory effects of Mn-doped biomaterials have attracted much attention due to the roles of Mn in nutritional immunity [18,47]. In vitro studies afford the opportunity to simulate complex interactions among cells in relatively controlled environments. This work sought to investigate the cellular immunomodulation impact of Mn-implanted surfaces and its influence on the osteogenic differentiation of mBMSCs in vitro. Previous studies have reported that stimulation with Mn can significantly upregulate the expression of genes such as IFN-β in Raw264.7 cell lines, indicating manganese’s potential to activate the immune response [48]. However, it has been reported that the cytokine of Raw264.7 cells to stimulants was different from that of human leukocytes to a certain degree; thus, caution should still be taken when extending the results of this experiment to the immune responses related to human cells.

The roles of surface properties such as surface topography, wettability, and chemistry in osteogenic activities and macrophage polarization are well recognized [49,50]. Therefore, the characterization of the surface properties of Mn-implanted surfaces is essential to elucidate osteogenic activities and immunological responses. Although the amount of Mn implanted on the two surfaces could not be directly determined using XPS, the ICP-MS results showed that the concentration of Mn released from Mn120 was higher than that from Mn60, indicating that the amount of Mn implanted in Mn120 was larger than that in Mn60. It has been reported that the Ti-Mn system exhibits a pure Ti phase when the Mn content is below 50% [51]. After implantation, Mn may exist in the form of a solid solution in a titanium matrix. This explains why no visible changes in surface topography were observed [52]. Referring to the model established by Jones et al., the etching process and the deposition process of the modified layer could exist simultaneously during the PIII&D, and the amount of implanted ions on the surface layer is significantly less than that in the deep deposited layer within the modified range [53]. This could explain why the surface of Mn60, despite the presence of a certain deposition layer, showed more etching characteristics and a decreased elastic modulus compared to those of Ti and Mn120. More specifically, the disruption of the surface structure caused by the initial ion implantation prior to the formation of a sufficiently thick deposited layer may be considered as another influencing factor [54,55]. In addition, ion implantation may have produced residual stress on the surfaces, which resulted in higher surface nano-hardness. With the extended implantation time, the deposited layer of Mn120 may be thicker, leading to its obviously enhanced nano-hardness compared to that of Ti and Mn60 [55]. As demonstrated in previous studies, cells can sense the mechanical cues, such as elastic modulus, dimensionality, and pattern, and then control the function of cells [56,57]. It can be seen that Mn implantation had little effect on the adhesion and morphologies of Raw264.7 cells and mBMSCs (Figure 3b and Figure 4a); thus, the differences in surface elastic modulus and nano-hardness were considered to exert limited effects on cells. Figure 2e shows that Mn-implanted samples, especially Mn120, displayed a tendency progressing towards less negative zeta potential values (−24.3 mV) than Ti (−61.2 mV) at pH 7.4, which may lead to increased adsorption of negatively charged proteins in the culture medium and body fluids [58]. 

Previous studies have established that a low concentration of manganese is optimal for the modulation of immune cells, while excessive amounts can result in toxicity [59]. In this study, PIII&D has been employed to introduce a small amount of Mn to a Ti surface, which released Mn ions at a concentration of 0–10 ppb. 

Mn is an essential micronutrient required for diverse biological activities and has been found to exhibit potential osteogenic effects on bone mineralization [60,61]. However, this study found that Mn-implanted surfaces had little effect on the expressions of osteogenic-related markers at both mRNA level (ALP, COL, OCN, and Runx2) and protein levels (ALP activities, collagen secretion, and ECM mineralization) under mono-culture conditions. Therefore, it is proposed that this osteogenic effect may be indirectly mediated by the regulation of manganese when co-cultured with macrophages. As shown in Figure 5, the osteogenic-related markers were found to be significantly upregulated at both the gene and protein levels under the co-culture conditions. Given the complexity of the osteogenic process and in conjunction with other experimental data, it can be inferred that the overall results obtained are conducive to promoting cellular osteogenic activities. Therefore, further investigation is warranted to fully comprehend the mechanisms underlying the changes produced by macrophages under co-culture conditions, particularly the role of Mn in this process.

Macrophages play a crucial role in the immune response and are crucial mediators of tissue homeostasis and remodeling [7,62]. In this study, a small number of Mn-doped surfaces exhibited good biocompatibility with Raw264.7 cells and could stimulate the polarization of Raw264.7 cells towards the M1 phenotype by stimulating the secretion of iNOS (M1 marker) and upregulating the mRNA expressions of pro-inflammatory cytokines (TNF-α) and chemokines (CCL3, CCL5, CSF2) under mono-culture conditions. Chemokines are found to exert a great effect on modulating the recruitment of immune cells, such as macrophages, which can clear cellular debris and promote healing [63]. For example, Mn can promote the recruitment and activation of monocytes to macrophages by increasing the gene expression of CCL3 [64,65]. Polarization and secreted cytokines of macrophages have been identified as important factors regulating tissue vascularization, which plays a key role in the process of bone development and remodeling [66]. Elevated exogenous Mn ions were proposed to increase the activity of Mn-SOD, which can help alleviate the oxidative stress state of endothelial cells and restore impaired angiogenic function [67]. The Mn-induced upregulation of specific chemokines, such as CSF2, can foster blood vessel formation around the implant, providing a source of oxygen and nutrients for the cells responsible for bone formation [68]. Additionally, the Mn-induced inflammation may cause a remodeling of the lymphatic network, which can form a versatile transport network to promote cellular osteogenic activities [66]. Therefore, inflammation at a low-grade level may be beneficial for osteogenic activities. 

Different from the pro-inflammatory immune microenvironment constructed in mono-culture conditions, the co-cultured macrophages on Mn-implanted surfaces exhibited a more prominent M2 phenotype compared to those on the Ti surface (Figure 6c). Although BMSCs have been revealed to transform macrophages to the M2 phenotype by their interaction via direct and indirect contact, Mn-implanted surfaces significantly enhanced the mRNA expression of anti-inflammation markers including IL-4, IL-10, CD206, and Arg-1 while downregulating pro-inflammatory marker TNF-α and chemokines such as CCL3, CCL5, and CSF2 (Figure 6c). Comparatively speaking, macrophages were more sensitive to Mn at a low concentration (0–10 ppb) than mBMSCs. After co-culture, the presence of mBMSCs and manganese in the environment together promoted the transformation of macrophages from the M1 to the M2 phenotype. This work shows an enhanced osteogenic differentiation of mBMSCs during a 10-day culture period. Nonetheless, since bone remodeling is a long-term process and the phenotypic shift of mBMSCs and macrophages occurs during that process, it may be necessary to conduct experiments over shorter or longer periods to refine the mechanism of the crosstalk between the two types of cells [4]. Moreover, the in vitro result also needs to be more rigorously validated by in vivo animal experiments in the future. Given these findings, it is believed that there is further potential for the application of Mn-doped orthopedic implants.

## 5. Conclusions

In this study, we utilized the PIII&D technique to successfully introduce Mn onto the surface of titanium plates and characterized the changes in the physicochemical properties. Mn-implanted surfaces, especially Mn120, exhibited elevated surface zeta potentials (pH = 7.4) and enhanced surface nano-hardness (within 110 nm). Although Mn implantation stimulated M1 phenotypes of macrophages and showed little effect on mBMSCs under mono-culture conditions, it can improve cellular osteogenic differentiation by mediating the crosstalk between mBMSCs and macrophages under co-culture conditions. Therefore, Mn-modified materials showed the potential to promote bone formation through immunomodulation.

## Figures and Tables

**Table 1:** The primary parameters used in the plasma ion immersion implantation.

Parameters	Mn60	Mn120
Target voltage pulse duration (μs)	500	500
Cathodic arc voltage pulse duration (μs)	800	800
Pulsing frequency (Hz)	5	5
Voltage (kV)	−15	−15
Time (min)	60	120
Pressure (Pa)	5.0 × 10^−3^	5.0 × 10^−3^

**Table 2:** The elemental contents of Mn-implanted samples.

Sample Name	C 1s (at%)	O 1s (at%)	Ti 2p (at%)	Mn 2p (at%)
Mn60	56.97	32.12	7.71	3.20
Mn120	51.99	37.01	6.91	4.09

**Table 3:** Corrosion potentials and currents of various samples.

Sample Name	Ti	Mn60	Mn120
*I_corr_* (A·cm^−2^)	3.387 × 10^−8^	4.235 × 10^−8^	2.997 × 10^−8^
*E_corr_* (V) vs. SCE	−0.292	−0.309	−0.327

## Data Availability

Not applicable.

## Supplementary Materials

The following supporting information can be downloaded at https://www.mdpi.com/article/10.3390/jfb14090456/s1, Figure S1: Schematic of the co-culture experiment (Raw264.7 cells with mBMSCs); Table S1: Primers used in RT-qPCR assays.
