# Supplementary material for: Manganese-Implanted Titanium Modulates the Crosstalk between Bone Marrow Mesenchymal Stem Cells and Macrophages to Improve Osteogenesis
Source: J Funct Biomater. 2023 Sep 3;14(9):456. doi: 10.3390/jfb14090456 (PMC10531852; doi:10.3390/jfb14090456)
Supplement: Supplementary file 1 [file jfb-14-00456-s001.zip › jfb-2578300-supplementary.pdf]

Manganese-implanted Titanium Modulates the Crosstalk between BMSCs and Macrophages to Improve Osteogenesis

Kuicai Ye [1,2], Xianming Zhang [1], Li Shangguan [1,3], Xingdan Liu [1,2]Xiaoshuang Nie [1,2], and Yuqin Qiao [1,*]

[1] *State Key Laboratory of High Performance Ceramics and Superfine Microstructure, Shanghai Institute of Ceramics, Chinese Academy of Sciences, Shanghai 200050, China*

[2] *Center of Materials Science and Optoelectronics Engineering, University of Chinese Academy of Sciences, Beijing 100049, China*

[3] *School of Materials Science, Shanghai University, Shanghai 200444, China*

* Correspondence: qiaoyq@mail.sic.ac.cn (Y.Q.)

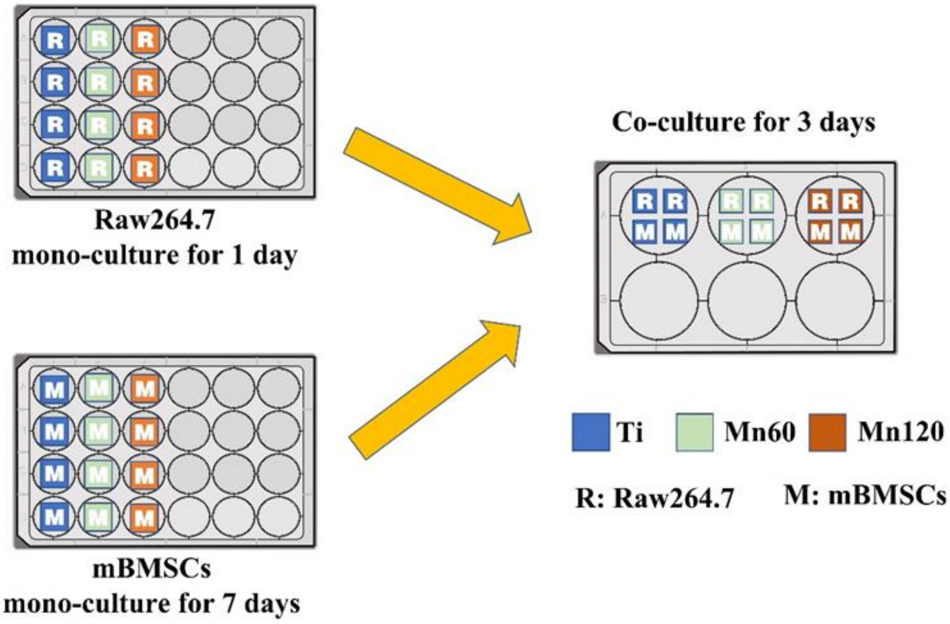

Figure S1. Schematic representation of the co-culture experiment (Raw264.7 with mBMSCs).

Table S1. Primers used in RT-qPCR assays

Gene (mouse)	Primer sequences (F, forward; R, reverse; 5′-3′)
GAPDH	F: AAA TGG TGA AGG TCG GTG TG<br>R: AGG TCA ATG AAG GGG TCG TT
CCL3	F: GGA TAC AAG CAG CAG CGA GT<br>R: GCA GTG GTG GAG ACC TTC A
CCL5	F: CAG AGA AGA AGT GGG TTC AAG A<br>R: CGA GTG GGA GTA GGG GAT TA
CSF2	F: GCG TAA TGA GCC AGG AAC TTG<br>R: TGA GTC AGC GTT TTC AGA GGG
TNF-α	F: TAG CCA GGA GGG AGA ACA GA<br>R: CCA GTG AGT GAA AGG GAC AGA
CD206	F: AGG GAA GAG AAG AAG ATC CAG<br>R: TGG GAG AAG ATG AAG TCA AAC
Arg-1	F: GCC AGG GAC TGA CTA CCT TAA<br>R: AGT TCT GTC TGC TTT GCT GTG
IL-4	F: TCA TCC TGC TCT TCT TTC TCG<br>R: CTT CTC CTG TGA CCT CGT TCA
IL-10	F: AGT GTG TAT TGA GTC TGC TGG<br>R: GAG AGA GGT ACA AAC GAG GTT
ALP	F: GCA GGC AAG ACA CAG ACT<br>R: TGG AGG AGA GAA GGT CAG AT
RUNX-2	F: GCA GCA CGC TAT TAA ATC CAA<br>R: GCC AAA CAG ACT CAT CCA TTC
OCN	F: ACC GCC TAC AAA CGC ATC TA<br>R: AGA GGA CAG GGA GGA TCA AGT
COL	F: TGA CTG GAA GAG CGG AGA GTA<br>R: GAC GGC TGA GTA GGG AAC AC
